# Extended Versus Standard Pouch in Roux-en-Y Gastric Bypass: Five To Nine Year Follow-Up Results of a Randomized Controlled Trial

**DOI:** 10.1007/s11695-026-08528-1

**Published:** 2026-02-13

**Authors:** Mitchell J. R. Harker, Sietske Okkema, Maud Schuurman, Laura Heusschen, Guusje Vugts, Eric J. Hazebroek

**Affiliations:** 1 Vitalys obesity clinic, part of Rijnstate Hospital, Arnhem, Netherlands; 2 Division of Human Nutrition and Health, Wageningen University, Wageningen, Netherlands; 3 Gelderse Vallei Hospital, Ede, Netherlands

**Keywords:** Metabolic bariatric surgery, Roux-en-Y gastric bypass, RYGB, Extended pouch Roux-en-Y gastric bypass, Long-term follow-up

## Abstract

**Background:**

Metabolic bariatric surgery (MBS) such as the Roux-en-Y gastric bypass (RYGB) is effective in the treatment of obesity. However, not every patient achieves optimal clinical response and recurrent weight gain remains a concern. Hypothetically, a narrow longer pouch could lead to better results by preventing pouch dilatation and slowing down gastric emptying rates. The aim of this study is to evaluate the effect of an extended pouch gastric bypass (EP-RYGB) on weight loss and quality of life 5 to9years (median 109 months [104–116]) postoperatively.

**Methods:**

Follow-up study of a single-blinded RCT including 62 patients who underwent a standard Roux-en-Y gastric bypass (S-RYGB, *n* = 30) versus EP-RYGB (*n* = 32) between September 2014 and October 2015. Outcomes on weight loss, obesity related complications, health-related quality of life (HRQoL), and gastro-intestinal symptomswere compared between S-RYGB and EP-RYGB.

**Results:**

Mean total weight loss (%TWL) was higher in EP-RYGB compared to S-RYGB (26.1 ± 11.2%, versus 24.1 ± 10.1%) although not statistically significant. More patients in the S-RYGB group tended to experience recurrent weight gain compared to EP-RYGB (70% versus 47%, *p* = 0.07). HRQoL and gastro-intestinal symptoms were comparable between groups (*p* > 0.05 for all).

**Conclusion:**

EP-RYGB results in slightly better weight loss outcomes and similar HrQoL compared to S-RYGB 5 to 9 years postoperatively. However, due to loss to follow up, the current study is underpowered and a definitive long term advantage of EP-RYGB cannot be concluded.

**Supplementary Information:**

The online version contains supplementary material available at 10.1007/s11695-026-08528-1.

## Key Points

Extended Pouch Roux-en-Y Gastric Bypass showed a non-significant trend towards better long term TWL compared to Standard Roux-en-Y Gastric Bypass.

Patients who underwent Standard Roux-en-Y Gastric bypass were more prone to developing recurrent weight gain.

Health-related quality of life was similar for Standard versus Extend Pouch Roux-en-Y Gastric Bypass.

Due to loss of long term follow-up compared to the initial trial, the abovementioned key points should be interpreted with caution, since the required number of patients for > 80% of power was no longer reached.

**Supplementary Information:**

The online version contains supplementary material available at 10.1007/s11695-026-08528-1.

## Introduction

Metabolic bariatric surgery (MBS) has proven to be the most effective and durable treatment for severe obesity [1]. The Roux-en-Y gastric bypass (RYGB) is one of the most performed surgical procedures in the treatment of severe obesity accounting for 32% of all primary metabolic bariatric procedures worldwide according to the 9th global registry report by the International Federation of Surgery in Obesity (IFSO) [2]. The results of RYGB regarding weight loss are considered superior compared to sleeve gastrectomy (SG) [3]. However, RYGB still provides suboptimal results in a subset of patients. Around 23% of patients experience suboptimal clinical response after surgery defined as a total weight loss (TWL) of less than 20% by IFSO [4, 5]. Moreover, around 18% of patients experience recurrent weight gain after surgery, defined as a ≥ 30% increase of total weight lost in kilograms from nadir [6].

One factor under investigation in the context of suboptimal results after RYGB, is the size of the gastric pouch. Previous studies have suggested that a smaller pouch in absolute volume leads to better weight loss results [7, 8], while a larger pouch may be related to a higher risk of marginal ulcers [7–9], poorer weight loss outcomes and a higher incidence of dumping related complaints [7]. However, the amount of research conducted on this topic is limited, consisting mostly of observational studies with uncertain and inconsistent conclusions and an evident lack of long-term data in general. This is underscored by a systematic review conducted by Mahawar et al. (2020) [7], who identified only 14 studies on the effect of gastric pouch size on weight loss, with only two of them being randomized controlled trials.

At our clinic, a randomized controlled trial (RCT) was conducted between September 2014 and October 2015 to evaluate the effect of an extended gastric pouch of 10 cm in a Roux-en-Y gastric bypass (EP-RYGB) compared to a standard 6 cm pouch (S-RYGB) [10]. The underlying hypothesis, supported by LaPlace’s and Poiseuille’s laws, is that a longer, narrower pouch will dilate less, delay gastric emptying, and promote a stronger, sustained feeling of fullness compared to a shorter, wider pouch [11, 12]. These factors may help reduce recurrent weight gain. The three-year results of this trial showed a significant difference of 31% versus 27% in TWL, in favor of the EP-RYGB. This was caused by less recurrent weight gain in the EP-RYGB group.

The aim of this study is to present the five to nine year outcomes of the previously performed RCT in our clinic, comparing EP-RYGB versus S-RYGB, in order to determine whether extending the pouch is a gripping point to improve long-term weight loss outcomes and quality of life (QoL) after RYGB.

## Methods

### Study Design and Population

**Fig. 1:**
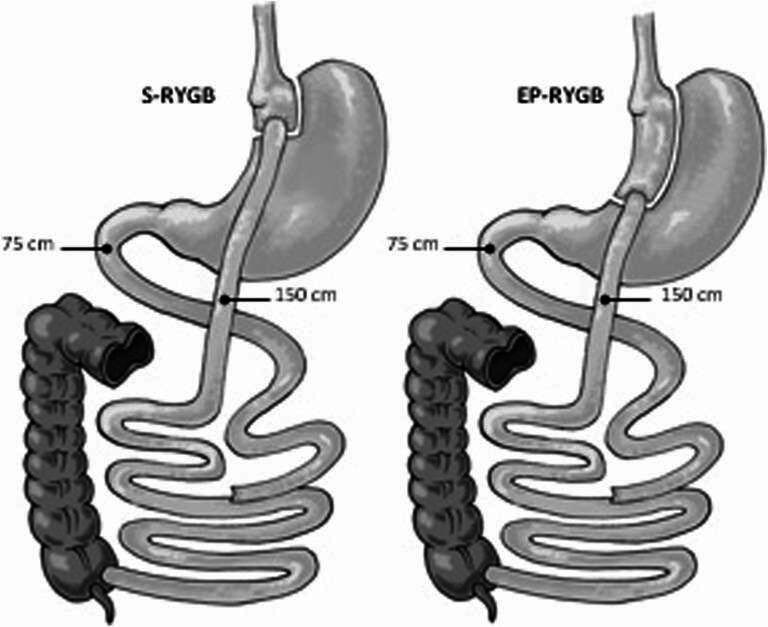
Anatomy of the surgical procedures. S-RYGB Standard Roux-en-Y gastric bypass, EP-RYGB Extended pouch Roux-en-Y gastric bypass

This study is a follow-up study of a single-blinded RCT conducted by Boerboom et al. [10]. In the initial trial, 134 patients were randomized into two groups: *n* = 65 in the S-RYGB group and *n* = 69 in the EP-RYGB-group. In the S-RYGB group *n* = 64 received the intervention, and *n* = 67 in the EP-RYGB group as randomized. The anatomy of both procedures is shown in Fig. 1.

To create the S-RYGB the first blue 60-mm lineal stapler (Echelon, Ethicon, Johnson & Johnson, New Brunswick, NJ, USA) was placed 5 cm below the angle of His at the right angle to the minor curvature of the stomach. The small proximal pouch was finished using two 60-mm staplers placed against a 40 French stomach tube. The EP-RYGB was created firing the first stapler 10 cm below the angle of His and was finished using three blue 60-mm staplers against a 40 French stomach tube.

For a detailed description of the RCT and the surgical procedures, we refer to the primary study [10]. The initial study had a follow-up period of 3 years and sample size calculation was based on the assumption that the EP-RYGB leads to 5% higher TWL after 2 years. Using a power of 80%, a sensitivity of 90%, a SD of 9.3%, and taking into account a drop out of 10%, a minimum of 65 patients per group were required.

The current study describes the five to nine years follow-up results of this trial. Additionally, patients were invited to complete additional questionnaires at nine years of follow-up (median follow-up time: 109 months [104, 116]). Patients were invited by email and provided with information about the study. Patients who did not respond were contacted by telephone and provided with information about the study. The questionnaires that were sent to the patients covered their current and nadir weight after surgery, the presence of obesity related complications, health-related quality of life (HRQoL) and symptoms of dumping syndrome.

In the initial trial, exclusion criteria were a history of previous MBS, patients who withdrew from participation before surgery, any form of inflammatory bowel disease (IBD), therapy resistant gastroesophageal reflux disease (GERD), and known pregnancy in the follow-up period. For this follow-up study, patients with a known pregnancy in the additional follow-up period (*n* = 1 in the S-RYGB group and *n* = 2 in the EP-RYGB group), patients who were deceased (*n* = 2 in the S-RYGB group and *n* = 2 in the EP-RYGB group), and patients who did not give consent for future research, withdrew from participation, or were lost to follow-up (*n* = 61).

The study protocol was reviewed and approved by the institutional review board of Rijnstate Hospital.

### Data Collection

Weight loss parameters up to five years postoperatively were obtained through patients’ electronic medical records. Nine year follow-up data on weight loss parameters, presence of obesity related complications, HRQoL and symptoms of dumping syndrome were obtained through questionnaires sent out to the participants via Research manager; Nova Business Software, Zwolle, The Netherlands.

Weight loss was defined as %TWL (weight loss at follow-up divided by preoperative weight). Recurrent weight gain was defined as a ≥ 30% increase of total weight lost in kilograms from nadir after RYGB.

Presence of the obesity related comorbidities type II diabetes mellitus (T2DM), hypertension, dyslipidemia and obstructive sleep apnea syndrome (OSAS) were compared at baseline and 109 months postoperatively. Additionally, total remission rates and newly developed obesity related complications were also analyzed.

HRQoL was measured with the BODY-Q questionnaire using the domains body image, physical activity, physical function, psychological function, sexual well-being and social function. Scores in the BODY-Q questionnaires range from 0 to 100 with 0 being the worst score and 100 the best [13]. Gastro-intestinal symptoms that were evaluated included GERD and dumping syndrome. Complaints of GERD were assessed using the GERD-Health Related Quality of Life Questionnaire (GERD-HRQoL), which contains ten questions concerning reflux and dysphagia. A total score of 0 is equal to no complaints and a score of 50 to very severe complaints [14]. Symptoms of dumping syndrome were assessed, using two questionnaires: Sigstad and Arts. The Sigstad questionnaire consists of sixteen questions about symptoms with a certain score per question. A score higher than seven is suggestive for dumping syndrome, and a score of less than four suggests an alternative diagnosis [15]. The Arts questionnaire is divided up into an early dumping score (with eight symptoms) and a late dumping score (with six symptoms). Each symptom was graded with: 0 for absent, 1 for mild, 2 for relevant and 3 for severe. This results in a maximum score of 24 for early dumping and 18 for late dumping [16].

### Data Analysis

Data are reported as mean ± standard deviation (normal distribution) or as median [Q1, Q3] (non-normal distribution) for continuous variables, and as frequency (percentage) for categorical variables, unless stated otherwise.

Differences in patient characteristics between both treatment groups were compared using independent samples t-tests, Mann–Whitney U tests and Chi-square tests for normal continuous data, non-normal continuous data and count data, respectively.

Differences in weight loss outcomes between the groups were analyzed using independent samples t-tests. Additionally, weight loss outcomes were analyzed using an ANCOVA to adjust for the baseline covariates, i.e., age, sex, preoperative BMI, and preoperative T2DM.

Differences in HRQoL and patient-reported gastrointestinal symptoms were analyzed using independent samples t-tests, Mann–Whitney U tests and Chi-Square tests, respectively.

All statistical analyses were performed using IBM SPSS Statistics 29 for Windows (IBM Corp., Armonk USA). A two-sided p-value below 0.05 was considered statistically significant.

## Results

**Fig. 2:**
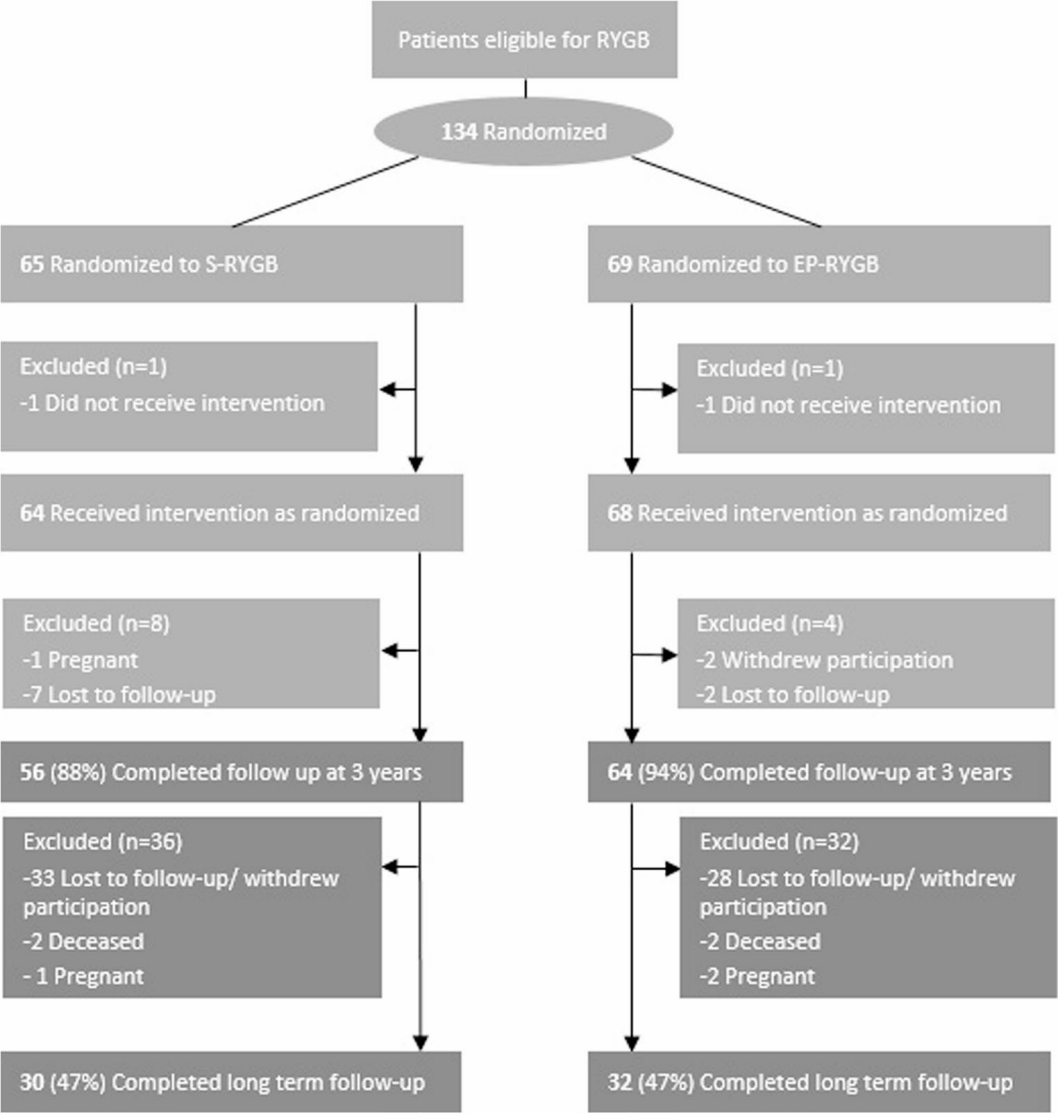
Flowchart diagram: number of patients during follow−up. S−RYGB Standard Roux−en−Y gastric bypass, EP−RYGB Extended Pouch Roux−en−Y gastric bypass

Between November 2023 and October 2024, of the 132 patients in the initial trial, 62 patients were reached and included for long term follow-up. This resulted in a follow-up rate of 47% for both groups (*n* = 30 in the S-RYGB group and *n* = 32 in the EP-RYGB group) **(**Fig. 2**)**.

The majority of the study population was female (74%) with a mean age of 47.2 ± 8.5 years.

(Table 1).

**Table 1:** Baseline patient characteristics for the total cohort, S-RYGB and EP-RYGB

	Total cohort (*n* = 62)	S-RYGB (*n* = 30)	EP-RYGB (*n* = 32)	*p* value
Gender (female)	46 (74.2)	21 (70.0)	25 (78.1)	0.57
Age (years)	47.2 ± 8.5	48.9 ± 6.7	45.5 ± 9.7	0.11
Weight (kg)	124.9 ± 18.7	125.7 ± 19.9	124.1 ± 17.7	0.73
BMI (kg/m^2^)	42.9 ± 4.6	43.0 ± 4.8	42.8 ± 4.4	0.87
Follow-up interval (months)	109 ± 7	109 ± 6	109 ± 7	0.97

Data represented as mean ± standard deviation, median [Q1, Q3] or frequency (%)

S−RYGB Standard Roux−en−Y Gastric Bypass, EP−RYGB Extended Pouch Roux−en−Y Gastric Bypass

Baseline characteristics including gender, age, preoperative weight, BMI, and follow-up interval. All baseline characteristics were comparable between the groups (*p* > 0.05 for all).

## Weight Loss

**Fig. 3:**
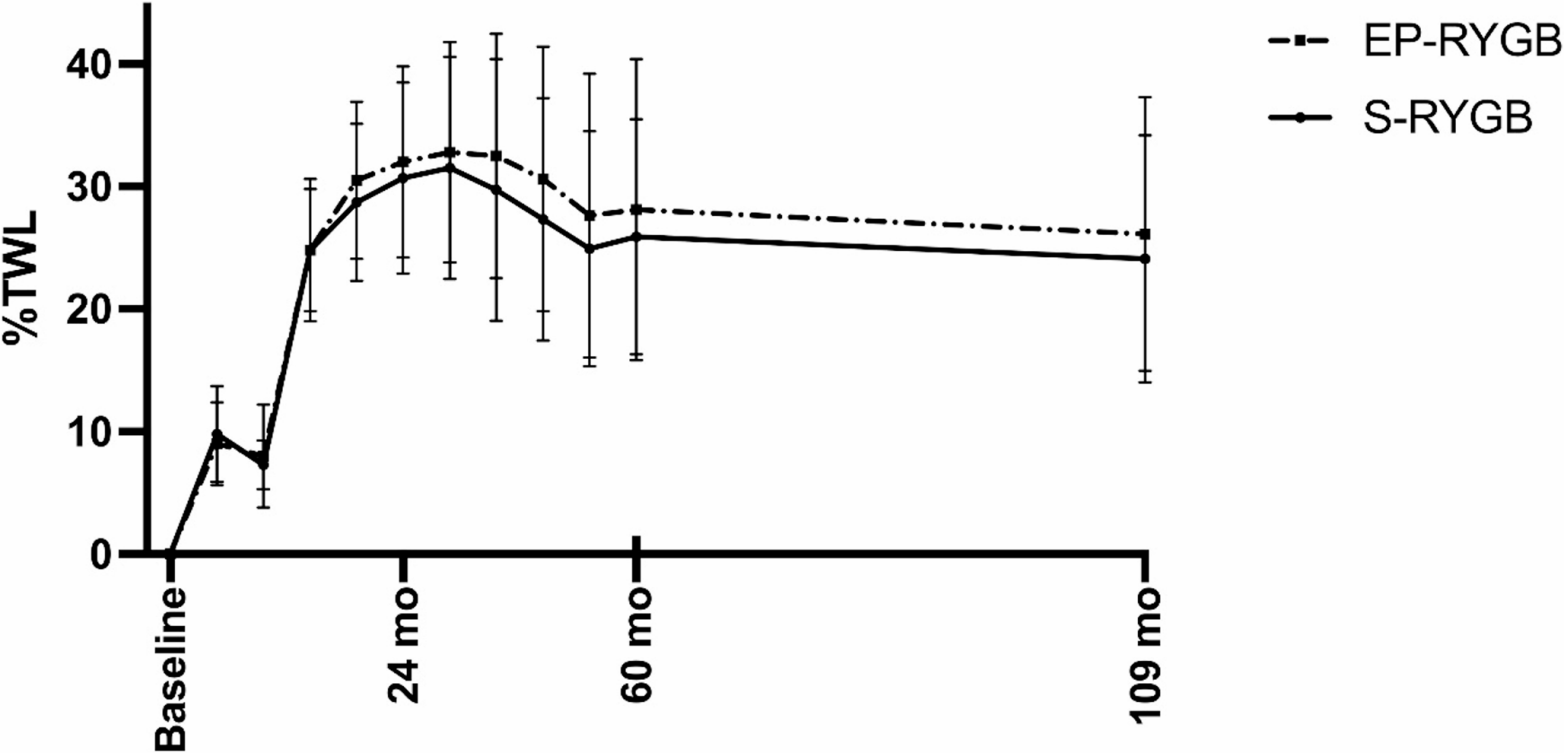
TWL (%) after S−RYGB and EP−RYGB. Data are presented as means ± standard deviation S-RYGB Standard Roux-en-Y gastric bypass, EP-RYGB Extended pouch Roux-en-Y gastric bypass

In the analysis of this follow-up study, no significant differences in terms of %TWL were found between the groups in both the crude and the adjusted analyses (*p* > 0.05 for all timepoints) (Table 2; Fig. 3).

**Table 2:** Weight loss outcomes

		*n*	Total cohort	*n*	S-RYGB	*n*	EP-RYGB	*p* value
Weight (kg)	Baseline	*62*	124.9 ± 18.7	*30*	125.7 ± 19.9	*32*	124.1 ± 17.7	0.73
	36 months	*60*	87.4 ± 16.5	*29*	90.5 ± 18.8	*31*	84.5 ± 17.7	0.16
	48 months	*54*	90.8 ± 17.7	*28*	92.9 ± 19.8	*26*	88.6 ± 15.1	0.38
	60 months	*55*	90.6 ± 16.6	*28*	93.1 ± 18.1	*27*	88.1 ± 14.9	0.28
	109 months	*62*	92.9 ± 16.2	*30*	94.9 ± 16.8	*32*	91.1 ± 15.6	0.36
BMI (kg/m^2^)	Baseline	*62*	42.9 ± 4.6	*30*	43.0 ± 4.8	*32*	42.8 ± 4.4	0.87
	36 months	*60*	30.1 ± 4.4	*29*	31.0 ± 4.7	*31*	29.3 ± 3.9	0.15
	48 months	*54*	30.7 ± 4.8	*28*	31.3 ± 5.0	*26*	30.0 ± 4.6	0.36
	60 months	*55*	31.0 ± 4.7	*28*	31.7 ± 4.8	*27*	30.1 ± 4.6	0.22
	109 months	*62*	32.0 ± 4.5	*30*	32.5 ± 4.7	*32*	31.4 ± 4.3	0.34
TWL (%)	Baseline	*62*	-	*30*	-	*32*	-	-
	36 months	*60*	29.0 ± 10.4	*29*	27.3 ± 9.9	*31*	30.6 ± 10.8	0.21
	48 months	*54*	26.2 ± 10.6	*28*	24.9 ± 9.6	*26*	27.6 ± 11.6	0.36
	60 months	*55*	27.0 ± 11.0	*28*	25.9 ± 9.6	*27*	28.1 ± 12.3	0.47
	109 months	*62*	25.1 ± 10.6	*30*	24.1 ± 10.1	*32*	26.1 ± 11.2	0.45

Data represented as mean ± standard deviation

S−RYGB Standard Roux−en−Y Gastric Bypass EP−RYGB, Extended Pouch Roux−en−Y Gastric Bypass

Maximum TWL at nadir was 34.6 ± 9.2% in the S-RYGB (median 18 [12, 17] months) and 35.5 ± 9.8% in the EP-RYGB group (median18 [12, 17] months] (*p* = 0.71), and 90% in the S-RYGB versus 91% in the EP-RYGB group had reached a TWL of ≥ 20%, postoperatively (*p* = 0.93).

Although not statistically different, more patients in the S-RYGB group tended to experience recurrent weight gain compared to the EP-RYGB group at nine years postoperatively; 70% versus 47%, respectively (*p* = 0.07).

### Obesity Related Complications

The presence of obesity-related complications at baseline and at nine years follow-up are shown in Table 3.

**Table 3:** Obesity-related complications

		S-RYGB (*n* = 30)	EP-RYGB (*n* = 32)	*p* value
T2DM	Baseline	13 (43.3)	8 (25.0)	0.13
	109 months	7 (23.3)	6 (18.8)	0.66
	Resolution	6 (46.2)	3 (37.5)	0.99
	Newly developed	0 (0)	1 (0.03)	0.99
Hypertension	Baseline	15 (50.0)	12 (37.5)	0.32
	109 months	7 (23.3)	5 (15.6)	0.44
	Resolution	10 (66.7)	8 (66.7)	0.99
	Newly developed	2 (6.7)	1 (3.1)	0.61
Dyslipidemia	Baseline	11 (36.7)	7 (21.9)	0.20
	109 months	3 (10.0)	4 (12.5)	0.99
	Resolution	9 (81.8)	4 (57.1)	0.33
	Newly developed	1 (3.3)	1 (3.1)	0.99
OSAS	Baseline	4 (13.3)	5 (15.6)	0.99
	109 months	0 (0.0)	4 (12.5)	0.11
	Resolution	4 (100.0)	1 (20.0)	**0.048**
	Newly developed	0 (0.0)	0 (0.0)	-

Data are represented as valid frequency (%)

S−RYGB Standard Roux−en−Y Gastric Bypass, EP−RYGB Extended Pouch Roux−en−Y Gastric Bypass, T2DM: Type II diabetes mellitus, OSAS: obstructive sleep apnea syndrome

At baseline, the S-RYGB group tended to have more patients with T2DM than the EP-RYGB group (13 (43.3%) vs. 8 (25.0%), *p* = 0.13. The resolution rates after 9 years were similar (46.2% vs. 37.5%, *p* = 0.99). A total of 27 patients (43.5%) were diagnosed with hypertension, of which 15 (50.0%) were in the S-RYGB group and 12 (37.5%) in the EP-RYGB group. The resolution rates after nine years did not differ (66.7% vs. 66.7%, *p* = 0.99). In total, 18 patients (29.0%) were diagnosed with dyslipidemia. In the S-RYGB group, 81.9% achieved resolution, compared to 57.1% in the EP-RYGB group (*p* = 0.326). Before surgery, 4 patients (13.3%) in the S-RYGB group suffered from OSAS, compared to 5 (15.6%) in the EP-RYGB group. The resolution rate was significantly different between the groups in favor of the S-RYGB (100% vs. 20%, *p* = 0.048).

## Health-Related Quality of Life

HRQoL was comparable between the groups for all domains of the BODY-Q questionnaires (*p* > 0.05 for all). On a scale of 0–100, highest scores regarding HRQoL were found in both S-RYGB and EP-RYGB for the subscales ‘physical activity’ (71 ± 23 vs. 73 ± 20), ‘psychological function’ (62 ± 28 vs. 66 ± 20), and ‘social function’ (67 ± 26 vs. 71 ± 23) **(**Table 4**)**.

**Table 4:** Health-related quality of life (BODY-Q) scores

	Total cohort (*n* = 61)	S-RYGB (*n* = 29) ^a^	EP-RYGB (*n* = 32)	*p* value
Body image	47 ± 29	46 ± 28	48 ± 30	0.80
Physical activity	72 ± 21	71 ± 23	73 ± 20	0.68
Physical problems	34 ± 6	34 ± 7	34 ± 6	0.82
Psychological function	64 ± 24	62 ± 28	66 ± 20	0.52
Sexual well-being	54 ± 30	52 ± 30	56 ± 30	0.64
Social function	69 ± 24	67 ± 26	71 ± 23	0.55

Data represented as mean ± standard deviation

a missing for n=1

GERD symptoms were low during long term follow-up at 60 (0.5 [0.0, 4.5] vs. 0.0 [0.0, 2.8]) and 109 months (0.0 [0.0, 5.5] vs. 0.0 [0.0, 5.0]). Maximum reported scores were 29 in the S-RYGB group and 35 in the EP-RYGB group, respectively. No significant differences were found at all timepoints mentioned (*p* > 0.05 for all).

The mean score of the Sigstad dumping syndrome questionnaire for both groups was similar (7.0 vs. 7.2, *p* = 0.915). Moreover, the percentage of participants that scored ≥ 7, did not significantly differ between the two groups either (48.3% vs. 40.6%, *p* = 0.61). For the Arts questionnaire, the S-RYGB group had a mean score of 4.3 for early dumping and 2.3 for late dumping, while the EP-RYGB group had a mean of 4.2 for early dumping and 2.3 for late dumping. These differences were not statistically significant (*p* = 0.90 and *p* = 0.99).

## Discussion

The aim of this study was to compare long term (5 to 9 years, median 109 months) outcomes on weight loss, obesity related complications, HRQoL and dumping syndrome between EP-RYGB and S-RYGB. Our data builds on the extended pouch trial of Boerboom et al. [10]. As mentioned earlier, according to Poiseuille’s Law, which states that flow rate is dependent on pipe length, patients with a longer pouch should have a longer pouch emptying time compared to patients with a short and wide pouch [11]. Theoretically, an extended pouch could result in slower pouch emptying and therefore induce a more gradual and longer period of gut hormone secretion resulting in a more pronounced metabolic effect of the RYGB. This was also stated by Deden et al., who found a slower gastric emptying rate in patients with optimal clinical response (TWL ≥ 20%) and a faster gastric emptying rate in patients with suboptimal clinical response (TWL ≤ 20%) [12]. Furthermore, Laplace’s law states that a long narrow pouch should have less tendency to enlarge compared to a short and wide pouch, which may also possibly explain the smaller proportion of recurrent weight gain in the EP-RYGB group [11]. According to this law, the narrower the tube, the lower the wall tension will be for the same internal fluid pressure. This emphasizes that the width of the pouch instead of the size determines its propensity to dilate over time [18].

Besides pouch dilatation, an enlarged stoma at the gastro-jejunal anastomosis could also contribute to recurrent weight gain. Especially the combination of an enlarged gastric pouch and stoma may result in a faster gastric emptying rate and loss of post-prandial satiety. Therefore, it has been stated that preventing this enlargement could improve results in the long term [19, 20]. Although stomal widening may be a contributing factor in the occurrence of recurrent weight gain, it is likely that this phenomenon was similar in the S-RYGB and EP-RYGB because creation of the gastrojejunostomy were performed identically.

Although our study showed a trend towards improved weight loss outcomes with EP-RYGB, these differences were not statistically significant during the postoperative period of 5 to 9 years.

(%TWL 24.1 ± 10.1 for S-RYGB versus 26.1 ± 11.2 for EP-RYGB). The initial study by Boerboom et al. [10] showed a significant difference in TWL at three years postoperatively. This significant difference is no longer visible in this study, possibly due to loss of follow-up and therefore a loss of power. In a systematic review by Mahawar et al. [7], fourteen studies were identified that describe the effect of gastric pouch and/or gastrojejunostomy size on weight loss outcomes in RYGB. Of these fourteen studies, five studies found that larger pouches to be associated with poorer weight loss outcomes, two of these studies found larger pouches in total volume to be associated with significantly lower weight loss and poorer diabetes outcomes. Furthermore, an additional nine studies were found that did not find any significant association between pouch size and weight loss outcomes. It is worth noting that two of these studies noted a non-significant trend in poorer weight loss outcomes with a larger gastric pouch in total volume. This renders our study the first in the existing literature to demonstrate a non-significant trend toward improved weight loss outcomes associated with a larger gastric pouch.

Interestingly, our study found a non-significant trend towards a higher prevalence of recurrent weight gain in the S-RYGB (70%) group versus EP-RYGB (47%). Heneghan et al. [19] examined the pouch length, width, volume and stoma diameter trough gastroscopy in patients with optimal clinical response (TWL ≥ 20%) versus patients that experienced recurrent weight gain. Their study found that pouch length and stoma diameter were significantly larger in the recurrent weight gain group. However, it is worth noticing that our study used a different definition of recurrent weight gain compared to the study of Heneghan et al. (≥ 30% increase of total weight lost in kilograms from nadir after RYGB versus a gain of ≥ 10 lbs from nadir after RYGB). Yimcharoen et al. [21]. also examined the pouch length, width, volume and stoma diameter trough gastroscopy and found pouch enlargement in 29% of patients that experienced recurrent weight gain. In their study, recurrent weight gain was also defined differently: weight gained after a patient’s nadir was reached after RYGB, respectively. This emphasizes the need for uniform reporting on definitions after MBS, so that results can be properly compared.

Remission rates of obesity related complications in our study were comparable between S-RYGB and EP-RYGB and high when compared to currently existing literature. For example, Sundbom et al. [22] described the resolution of obesity related complications ten years after primary RYGB using a nationwide registry including 29,578 patients. When comparing our remission rates to those of Sundbom et al., remission rates were respectively 43% vs. 30% for T2DM, 67% vs. 15% for hypertension, 65% vs. 9% for dyslipidemia and 38% vs. 4% for OSAS. A possible explanation for the high resolution rates of obesity related complications in this study is that patients were asked to self-report the presence or absence of obesity related complications and is not backed up by collection of plasma samples to determine serum values of for example glycated hemoglobin and cholesterol. Furthermore, it is important to emphasize the effect of MBS on T2DM. Mingrone et al. [23] showed a −0.9% (confidence interval (CI) −1.2 to −0.6) change in glycated hemoglobin, a −1.5 (CI −2.2 to −0.7) change in total number of DM medications used, and a 0.1 (CI 0.0 to 0.5) change in insulin use at ten years post RYGB compared to medical therapy for T2DM (*p* < 0.001 for all). Additionally, Axelrod et al. [24] showed that MBS profoundly altered the circulating metabolome causing important improvements in glucose homeostasis when compared to regular medical therapy for T2DM.

When assessing HRQoL, scores were acceptable and comparable between the groups for all domains of the BODY-Q questionnaires. To our knowledge, this is the first long term follow-up study that used the BODY-Q questionnaires to evaluate QoL in S-RYGB and EP-RYGB. Askari et al. assessed HRQoL using the Bariatric Analysis and Reporting Outcome System (BAROS), and found that MBS improves quality of live on the long-term at more than ten years after surgery [17].

Our study showed a low prevalence of GERD and low scores in the GERD-HRQoL questionnaire in both S-RYGB and EP-RYGB at 36, 60 and 109 months postoperatively. Unfortunately, the GERD-HRQol was not assessed preoperatively. Our results are comparable to a study by Salminen et al. [3], who found a median GERD-HRQoL score of 0.0 at ten years postoperatively versus 0.0 in this study.

When assessing the prevalence of dumping syndrome using the Sigstad questionnaire, our study showed that 48.3% in the S-RYGB versus 40.6% in the EP-RYGB group had scores that are suggestive for the presence of dumping syndrome. This suggests a potential advantage of EP-RYGB: the longer pouch and potentially slower gastric emptying may help explain the lower prevalence of dumping syndrome. To our knowledge, there is no other literature available on the long term prevalence of dumping syndrome using the Sigstad questionnaire. However, a study by Waridel et al. showed that up to 54% of patients experienced bothersome indigestion or abdominal complaints at ten years postoperatively [25]. In both S-RYGB and EP-RYGB, mean scores on the Arts questionnaire were low for early and late dumping. To our knowledge, no other literature describes long term outcomes after RYGB using the Arts questionnaire to assess early and late dumping.

### Strengths and Limitations

This study has several strengths. First, to our knowledge, this is the first available long term follow-up data from a RCT comparing S-RYGB with EP-RYGB. Previous studies on this subject were observational and descriptive in nature [8, 9, 26–30]. Second, the extensive description of health-related quality of life using the BODY-Q, GERD-HRQoL, Sigtad and Arts questionnaires gives important insights of patient reported outcomes following S-RYGB and EP-RYGB.

This study also has some limitations. First, the original study’s sample size was powered for a follow-up period of three years. As many other studies on MBS, we experienced a relatively high loss to follow-up rate. Despite al effort, we were only able to achieve a follow-up rate of 47% in both groups after a median of 109 months, resulting in a relatively small study population. However, there seems to be no selection bias present in this study; the baseline characteristics of the excluded and lost to follow-up patients versus the included patients are comparable. Second, follow-up data on weight, obesity related complications, and QoL at 109 months consists of self-reported patient data only and was not verified in a clinical or outpatient setting. Therefore, data on weight, obesity related complications, and QoL could be biased and form a concern in statistical reporting. Third, no radiographic studies nor 3D-CT-volumetry were performed to give any hard facts on the dilatation of the pouches over time. Therefore, the statement that stomal widening may be a contributing factor in the occurrence of recurrent weight gain remains hypothetical. Fourth, it is unknown if patients underwent other weight loss treatments (i.e. dietitian consultation, medication) during the follow-up period. If patients underwent other weight loss treatments, this could also have resulted in biased data.

In our opinion, more data on long term outcomes of metabolic bariatric procedures with sufficient sample size and adequate length of follow-up are needed. By addressing the aforementioned limitations, we are currently performing anew study ([removed for blind peer review].) comparing S-RYGB, EP-RYGB and banded extended pouch gastric bypass (BEP-RYGB) in a randomized cohort consisting of at least 120 patients per study arm.

## Conclusion

After nine years, EP-RYGB showed a trend towards better weight loss results compared to S-RYGB. Both techniques offer similar outcomes regarding remission of obesity related complications and quality of life improvement. S-RYGB tended to show more recurrent weight gain compared to EP-RYGB. Due to a high loss of follow-up, these findings should be considered exploratory, and definite conclusions on the long term advantages of EP-RYGB are subject of future studies with larger sample sizes.

## Supplementary Information

Below is the link to the electronic supplementary material.

Supplementary Material 1 (PPTX 2.79 MB)

## Data Availability

The data that support the findings of this study are available from the corresponding author upon reasonable request.
