# Supplementary material for: Extended Versus Standard Pouch in Roux-en-Y Gastric Bypass: Five To Nine Year Follow-Up Results of a Randomized Controlled Trial
Source: Obes Surg. 2026 Feb 13;36(3):1271–80. doi: 10.1007/s11695-026-08528-1 (PMC13038654; doi:10.1007/s11695-026-08528-1)
Supplement: Supplementary file 1 — Supplementary Material 1 (PPTX 2.79 MB) [file 11695_2026_8528_MOESM1_ESM.pptx]

## Slide 1
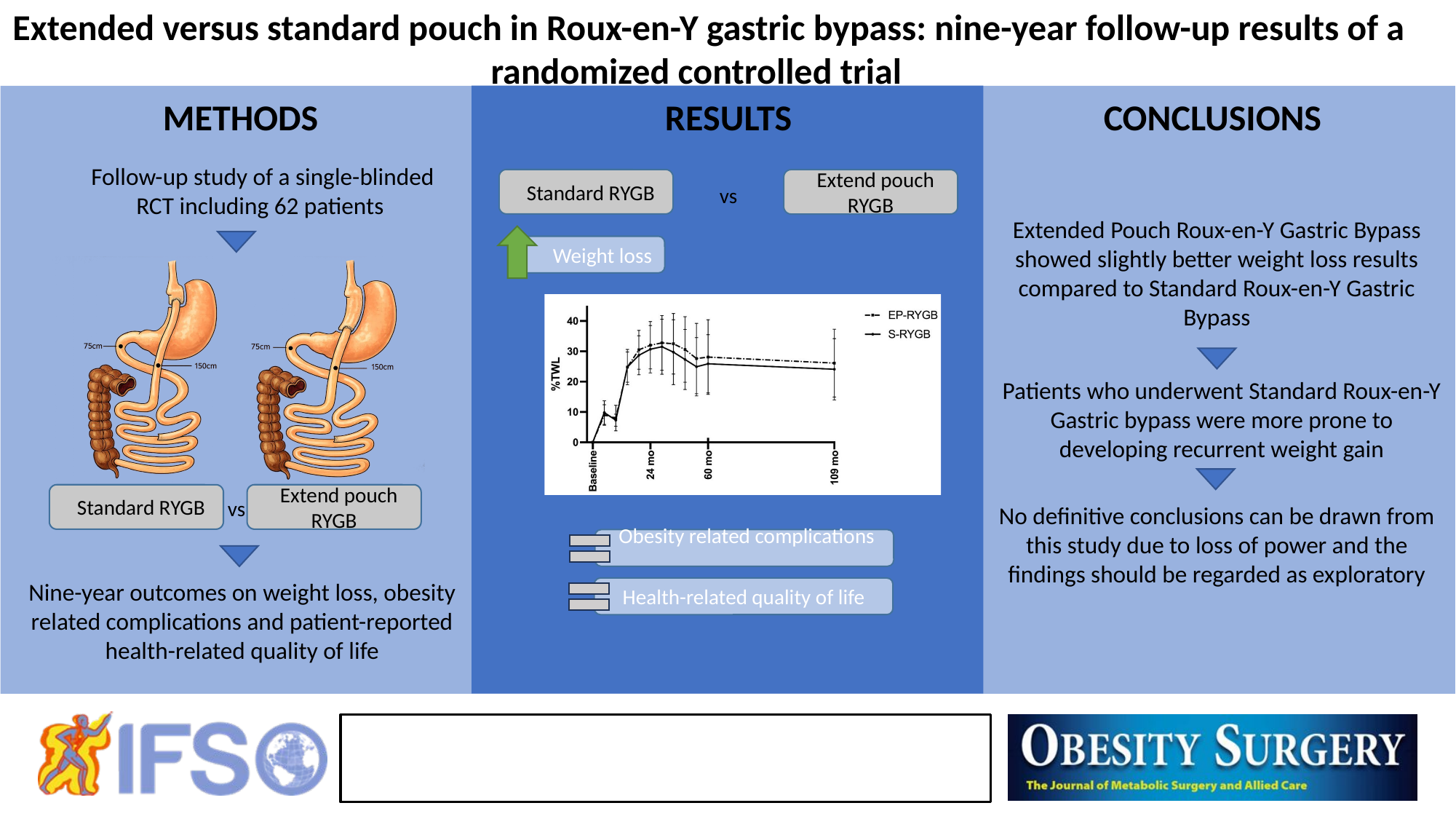

Extended versus standard pouch in Roux-en-Y gastric bypass: nine-year follow-up results of a randomized controlled trial
Conclusion
 METHODS
RESULTS
CONCLUSIONS
Follow-up study of a single-blinded RCT including 62 patients
 Standard RYGB
 Extend pouch RYGB
vs
Extended Pouch Roux-en-Y Gastric Bypass showed slightly better weight loss results compared to Standard Roux-en-Y Gastric Bypass
 Weight loss
Patients who underwent Standard Roux-en-Y Gastric bypass were more prone to developing recurrent weight gain
 Standard RYGB
 Extend pouch RYGB
vs
No definitive conclusions can be drawn from this study due to loss of power and the findings should be regarded as exploratory
 Obesity related complications
Nine-year outcomes on weight loss, obesity related complications and patient-reported health-related quality of life
Health-related quality of life
